# Resolving the Connectome, Spectrally-Specific Functional Connectivity Networks and Their Distinct Contributions to Behavior

**DOI:** 10.1523/ENEURO.0101-20.2020

**Published:** 2020-09-09

**Authors:** Robert Becker, Alexis Hervais-Adelman

**Affiliations:** 1 Neurolinguistics, Department of Psychology, University of Zurich, 8050 Zurich, Switzerland; 2 Neuroscience Center Zurich (ZNZ), 8057 Zurich, Switzerland

**Keywords:** canonical correlation analysis, connectome, networks, oscillations, resting state, variability

## Abstract

The resting human brain exhibits spontaneous patterns of activity that reflect features of the underlying neural substrate. Examination of interareal coupling of resting-state oscillatory activity has revealed that the brain’s resting activity is composed of functional networks, whose topographies differ depending on oscillatory frequency, suggesting a role for carrier frequency as a means of creating multiplexed, or functionally segregated, communication channels between brain areas. Using canonical correlation analysis (CCA), we examined spectrally resolved resting-state connectivity patterns derived from magnetoencephalography (MEG) recordings to determine the relationship between connectivity intrinsic to different frequency channels and a battery of over a hundred behavioral and demographic indicators, in a group of 89 young healthy participants. We demonstrate that each of the classical frequency bands in the range 1–40 Hz (δ, θ, α, β, and γ) delineates a subnetwork that is behaviorally relevant, spatially distinct, and whose expression is either negatively or positively predictive of individual traits, with the strongest link in the α-band being negative and networks oscillating at different frequencies, such as θ, β, and γ carrying positive function.

## Significance Statement

Even at rest, the human brain displays spontaneous coordinated rhythmic patterns of activity. Partitioning these according to their temporal properties reveals networks of distributed brain areas synchronized at different frequencies. The properties of these networks differ across individuals and are predictive of the brain’s response to tasks, pointing to a functional substrate underlying variability of task-related responses. The functional roles of these resting-state networks (RSNs) are yet to be fully elucidated. Here, in the absence of any task, we exploit the spectral richness of non-invasive magneto-encephalographic recordings to establish that individual differences in the expression of five spatio-spectrally distinct RSNs predict a diverse array of individual behavioral measures, from tobacco consumption to cognitive performance (CP).

## Introduction

Even in the absence of any task, the brain expresses patterns of functional connectivity (FC). Such resting-state activity is thought to be determined by intrinsic features of the underlying neural architecture (Fox and Raichle, 2007). Thus, spontaneous activity of the brain provides an insight into endogenous features, which are increasingly understood to be substantial contributors to individual differences in behavior. A number of recent studies have highlighted the extent to which resting-state connectivity metrics reveal individual “wiring patterns” of the brain that are significantly predictive of behavioral, and even demographic features. Notably, resting-state fMRI (rs-fMRI) connectivity patterns have been shown to be predictive of task-related activation patterns (Tavor et al., 2016) and even to constitute uniquely identifying fingerprints that can distinguish between individuals (Finn et al., 2015). Such findings suggest that resting-state activity patterns reflect a neurobiological framework that determines a large degree of individual differences in brain responses to tasks, and of individual differences in behavior. In a sense, they may be considered the brain’s “priors” (Kersten et al., 2004) for determining how it responds to input.

A recent effort to relate whole-brain rs-fMRI connectivity to individual differences in behavior employed canonical correlation analysis (CCA) to establish how interindividual differences in connectivity relate to differences in a broad battery of behavioral and cognitive variables (Smith et al., 2015), using a sample of 461 participants from the Human Connectome Project (Van Essen et al., 2013). CCA was used to find the maximal correlations between combinations of variables in the connectivity and subject measures, revealing a canonical mode of covariation that is highly positively correlated with positive cognitive traits as well as desirable lifestyle indicators. This is a fascinating finding that highlights the extent to which large-scale neural connectivity of the human brain, even at rest, is functionally relevant. However, this discovery invites further and deeper inquiry: fMRI is rightly prized for its high spatial resolution, but its temporal resolution is inherently limited by the sluggishness of the hemodynamic response that it records. Consequently, fMRI cannot be used to discriminate between neural processes that occur at timescales in the subsecond range. These are, however, of profound relevance to further elucidating the nature of resting-state brain activity and its relationship to behavior.

A well-known characteristic feature of the human brain’s electrical activity at rest is the presence of oscillatory signals (Berger, 1929; Adrian and Matthews, 1934; Pfurtscheller, 1981). Oscillations are the product of repetitive or cyclical patterns of brain activity, which are observed to occur at different frequencies. Oscillations exist not only at multiple temporal scales, but also spatially they range from single neuron membrane potential oscillations, to local field potentials and large-scale magneto-electric brain signals. Temporally, different oscillatory frequencies are thought to represent different channels in which neural activity is communicated, and might therefore fulfil different functional roles (Bastos et al., 2015). Even at a relatively coarse level of spatial resolution, an extensive literature of M/EEG or electrophysiological studies has established how neuronal processes occurring in different frequency ranges have different functional associations, from the relatively slow sleep rhythms (e.g., δ 1- to 3-Hz waves in NREM sleep; de Andres et al., 2011) to memory processes in the hippocampus (Buzsaki, 2002; Cantero et al., 2003; mostly in the 4- to 7-Hz θ range), to processes related to sensation (covering the α and β range of 8–30 Hz; Berger, 1929; Pfurtscheller, 1981) or executive functions such as working memory (Klimesch et al., 1997; Sauseng et al., 2002; Hanslmayr et al., 2016), up to rhythms in higher frequencies such as γ (30 Hz and above), involved in feature binding during visual and other task processing (Engel et al., 1991; Fries, 2015).

Oscillations can be characterized in terms of frequency, amplitude and phase. Much of the research into the functional relevance of human neural oscillations has focused on linking properties of ongoing rhythms such as phase (Callaway and Yeager, 1960; Dustman and Beck, 1965; Busch et al., 2009; Mathewson et al., 2009; Varela et al., 2020) or amplitude (Jasiukaitis and Hakerem, 1988; Rahn and Basar, 1993; Makeig et al., 2002; Nikulin et al., 2007; Becker et al., 2008; Mazaheri and Jensen, 2008; Reinacher et al., 2009), to behavioral and perceptual performance in a variety of experimental task conditions, domains and sensory systems or to modulated task responses. When also combined with multimodal imaging this amplitude or phase-informed approach has also shown that rhythms index different states of neuronal excitability (Goldman et al., 2002; Laufs et al., 2003a,b; Moosmann et al., 2003; Becker et al., 2011; Haegens et al., 2011; Scheeringa et al., 2011).

However, while there is consensus that large-scale rhythms such as α or β rhythms and their functional relevance (as mentioned above) are emergent properties of an interconnected network of neuronal ensembles (Speckmann and Elger, 2005), a proper network-centric view of these large-scale oscillations is a relatively new approach in M/EEG research, and often M/EEG studies examining functional relevance of rhythms have conducted analyses that elucidate relationships at the sensor or source level (for a non-exhaustive list of examples see Klimesch, 1999; Altenmueller et al., 2005).

Adopting a network-based view in the context of M/EEG imaging is particularly attractive given the high temporal resolution of the method, opening up new possibilities in terms of exploiting the rich spectro-temporal information that can be derived from M/EEG recordings. This adds an entire new dimension to the analysis of functional networks, namely the frequency these networks are operating at, which, in the mammalian brain can span ranges from ultraslow (<0.1 Hz) to ultrafast (>100 Hz) frequency regimes (Buzsáki et al., 2013). By examining the whole-brain networks defined by synchronous resting state in delineated frequency ranges, we can shed light simultaneously on not only the relationships between oscillatory frequency and behavior but also how different oscillatory networks are spatially organized (Sadaghiani and Wirsich, 2020).

Generally, synchronized patterns of modulation of activation at separate loci are believed to reflect coupled activation (Speckmann and Elger, 2005) and consequently to reveal connected areas, ultimately constituting functional networks. This would (technically) allow in M/EEG to look at these functional networks in different frequency regimes. However, what is not known is how meaningful these spectrally resolved networks are. Initial evidence for the multispectral nature of M/EEG connectivity is provided by findings from resting-state activity that highlight the existence of spectrally resolved large-scale brain connectivity networks (Brookes et al., 2011a,b; Hillebrand et al., 2012; Hipp et al., 2012; Sadaghiani and Wirsich, 2020). Recent efforts have identified large-scale (i.e., whole-brain) electrophysiological networks with topographical and spatial structures comparable to the resting-state networks (RSNs) established by fMRI studies. For example, motor or visual RSNs, analogous to those previously described in fMRI (Biswal et al., 1995; Damoiseaux et al., 2006) have been reported in magnetoencephalography (MEG; Brookes et al., 2011a,b; Hillebrand et al., 2012). Interestingly, these networks, while spatially similar to fMRI-derived RSNs, had distinct spectral properties. That is, they exist as coherent activity in different frequency regimes: the motor network being most pronounced in the β range (14–30 Hz), whereas networks incorporating visual areas were most visible in the α range 8–12 Hz. The finding of different topographies in spectrally distinguished networks hints at the potentially different function these spontaneous networks might serve, but beyond the more intuitively interpretable sensory-related and motor-related networks mentioned above, the roles and particular functional relevance of the spatial networks that emerge at different frequencies, remains elusive (see, for example, discussion in Sadaghiani and Wirsich, 2020). At this point, it is worth highlighting that all neural communication is constrained by the underlying anatomic connectivity, and that any network of information transfer that is discovered is necessarily a reflection of intrinsic brain architecture (“Structure defines function,” Buzsaki, 2006). Consequently, any relationship elucidated between FC patterns and behavior depends on neuroanatomy, but they are not fully determined by it (Uddin, 2013). One indicator of this view is the existence of connectivity in different frequency ranges which, if anything but noise, is suggestive of the possibility that the anatomically constrained connections can be exploited to multiple ends.

The precise role of network communication at different frequencies is the subject of ongoing and increasing interest. One overarching notion is that of “multiplexing,” namely, that different oscillatory frequencies comprise different communication channels allowing the same anatomic connections to be used for different functional roles (Akam and Kullmann, 2014). An influential theory with a very broad scope, communication through coherence (CTC), proposes that the observed spectrally-resolved networks, i.e., networks that share a common rhythm on a carrier frequency, reflect activity of cell assemblies that are in communication with each other, e.g., when binding different sensory modalities together (Fries, 2015). Coherence in the faster γ-band (40 Hz and above) in particular, is well examined and seems to confirm the proposals of CTC theory. CTC also affords the existence of similar mechanisms in other frequency bands, although these are less well explored and less is known about their likely functional role or roles.

Another recent focus of enquiry underscoring the importance of neural activity in different frequency bands, has been how different frequencies reflect the activity of neurons having different roles in the directionality of neuronal communication. For example, it is known that bottom-up processes are preferentially mediated by higher-frequency (e.g., γ) activity, while top-down processes have principally been associated with rhythms in lower frequencies (Bastos et al., 2012). In sum, there is compelling evidence indicating that spectrally confined, oscillatory activity may serve different purposes in network communication, be it in directing information flow or in circumscribing the neural assemblies that preferentially operate as coherent ensembles.

Despite these advances, a holistic analysis of the functional role of oscillatory networks, taking into account the full spectral richness of the underlying constituents of human spontaneous brain activity, and its relationship to an extensive range of behavioral factors has, so far, been beyond our reach. Here, we seek to expand on and complement the existing rs-fMRI findings by exploring the relationships between the same set of Subject measures and spectrally-resolved connectomes derived from rs-MEG, also made available as part of the HCP dataset (Larson-Prior et al., 2013).

Specifically, we address the following questions. First, can we identify and characterize a global mode linking brain connectivity and behavior using spectrally resolved MEG? Second, is this mode comparable to that revealed for rs-fMRI? And thirdly, what, if any, additional information can we glean from the spectral richness afforded by the high temporal resolution of MEG? By exploring these questions, we will shed light on the spatial structure and functional contributions of the spectral components of resting-state connectivity and how they relate to a broad spectrum of behavioral indicators.

## Materials and Methods

### Subjects and data

rs-MEG data from the Human Connectome Project were used for this study. A total of 89 subjects had complete resting-state recordings (i.e., three recording sessions of ∼6 min). Mean age: 29 ± 4 years, sex distribution: 41 female/48 male. Subjects were recorded in supine position and instructed to remain relaxed, with eyes open.

### Subject and behavioral measures

Following along the lines of a previous study showing CCA-derived brain-behavior mode with fMRI (Smith et al., 2015), we extracted a large number of subject measures from the HCP dataset. We excluded gender related subject measures and structural measures, but kept all other measures [cognitive, emotional, sensory performance tests, psychiatric and personality tests, family history of mental or other disorders, consumption of alcohol, tobacco or other drugs, in-scanner (fMRI) task performance]. From those, we excluded variables that did not fulfil the following criteria: (1) <80% of values in a subject measure should have same discrete values; (2) at least 49 out of 89 subjects should have non-missing values for a given subject measure (compared to previous studies; Smith et al., 2015, we chose this conservative number since our dataset is considerably smaller); and (3) the maximum value of any given value across subject measures should not exceed 100 times the mean of the group.

These exclusion criteria resulted in 131 behavioral variables that were used for further analysis.

We also deconfounded, i.e., regressed out, effects of age, handedness, gender, height, blood pressure (systolic and diastolic) and brain volume for all subjects to remove connectivity related effects mediated by these that might distort and confound connectivity measures (independent of the imaging modality, i.e., MEG or fMRI, since both were used in the control analyses). Finally, all variables were further subjected to rank-based inverse normal transformation to ensure Gaussianity of distributions and demeaned. The general approach followed preprocessing of behavioral variables as previously conducted for an fMRI-derived brain-behavior mode (Smith et al., 2015).

### MEG preprocessing and parcellation, source leakage correction

For MEG acquisition, MEG data were recorded by a whole-head MAGNES 3600 (4D NeuroImaging). Data were acquired in three runs of resting-state recordings, lasting 6 min each. The MEG recorded from 248 magnetometer sensors, with 23 reference channels. Sampling rate was at 508.63 Hz, and data were down-sampled for further processing to 200 Hz. The standard preprocessing pipeline as offered by the HCP (“tmegpreproc”) was used which applied independent component analysis to remove potential artefacts from ocular, muscular or cardiac sources. For source reconstruction, single shell volume models were used, based on the individual anatomic MRI T1 images provided by the HCP consortium. Linearly constrained minimum variance (LCMV) beamforming was employed onto a regular 3D grid in normalized MNI source space with a resolution of 8 mm^3^ using normalized lead fields and data covariance estimated in the 1- to 48-Hz broadband frequency range. Source activity was normalized by the power of the projected sensor noise. Using principal component analysis (PCA), the first principal component at each 3D source voxel location was extracted resulting in 5798 (1D) source-voxels. These were parcellated into 100 source parcels using a semi-data driven parcellation approach. Parcels were derived on the basis of a 246-region anatomic brain atlas (Jiang, 2013) that covers the two hemispheres (including subcortical structures). However, at this resolution, parcels are sometimes too closely related, resulting in rank deficiencies in the covariance matrix which in turn makes the following source leakage correction infeasible. Reducing them to 100 parcels averts this problem and offers a good compromise between spatial resolution and robustness for subsequent analysis. In order to obtain an optimal (maximally independent) reduced set of parcels from the original atlas^73^, we performed a k-means clustering of group (and session) average correlation matrices of the raw time series (parcellated into the initial 246 parcels per subject and session). This clustering identified parcels that were most correlated the most (in absolute terms) and these were subsequently merged, resulting in a final, set of 100 parcels with minimum correlation to each other. Parcellation at higher dimensionality, e.g., with *N* = 200, led to failure of the multivariate source leakage correction, which requires full rank covariance matrices. This apparent rank deficiency suggested that 200 parcels represents an overparameterization of the data, i.e., they data contain there are fewer than 200 independent dimensions. A visualization is provided in Extended Data Figure 1–1 (for a labeled list of parcels, see Extended Data Fig. 1-2).

**Figure 1.**
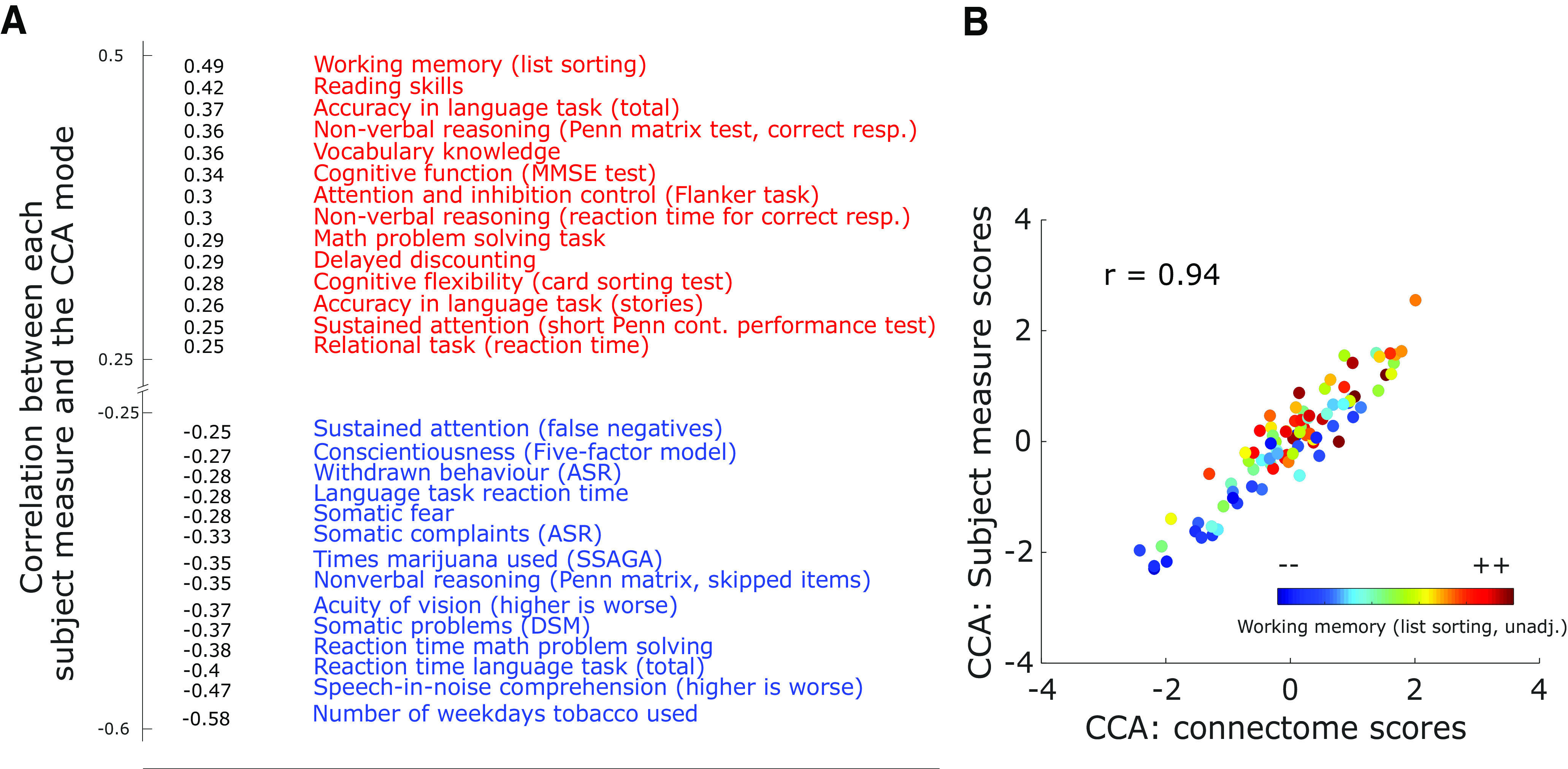
CCA of spectrally resolved MEG data and a large set of behavioral and other subject measures results in one significant mode (*r* = 0.94, *p* < 10^−4^, corrected for multiple comparisons by permutation testing). For the used parcellation, see Extended Data Figure 1-1, with anatomic labels found in Extended Data Figure 1-2. A visualization of the analysis approach is shown in Extended Data Figure 1-3. ***A***, The canonical mode arranges behavioral variables and subject measures on a positive-negative axis, similar to what has been previously reported for hemodynamic measures of brain connectivity (Smith et al., 2015; for comparison, see also Extended Data Fig. 1-4, showing the fMRI-based brain-behavior mode for the set of subjects used here). At maximum positive correlation, there are mostly subject measures indexing CP such as reading skills and vocabulary knowledge, while on the negative end of the spectrum are subject measures like somatic problems, and tobacco consumption (thresholded at a correlation coefficient of |*r*| > 0.25). ***B***, Correlation of CCA-derived subject measure scores and connectivity scores for the first canonical variates identified, i.e., the first canonical mode. In color the behavioral score for the working memory test is shown per subject. For a detailed overview of how certain methodological choices impact results see Extended Data Figure 1-5. The first CCA mode as visualized here also explains a significant amount of variance in the data (Extended Data Fig. 1-6). Penn matr. = Penn matrix test; ASR = Achenbach Adult Self Report; DSM = Diagnostic and Statistical Manual; MMSE = mini mental state examination; SSAGA = Semi-Structured Assessment for the Genetics of Alcoholism; unadj = unadjusted for age effects. Please note that some secondary measures (e.g., similar metrics for tobacco consumption) are left out to avoid redundancy.

10.1523/ENEURO.0101-20.2020.f1-1 Extended Data Figure 1-1 Visualization of the used parcellation (*n* = 100, lateral view). Parcel identities can be found in Extended Data Figure 1-2, with anatomical labels. Download Figure 1-1, TIF file.

**Figure 2.**
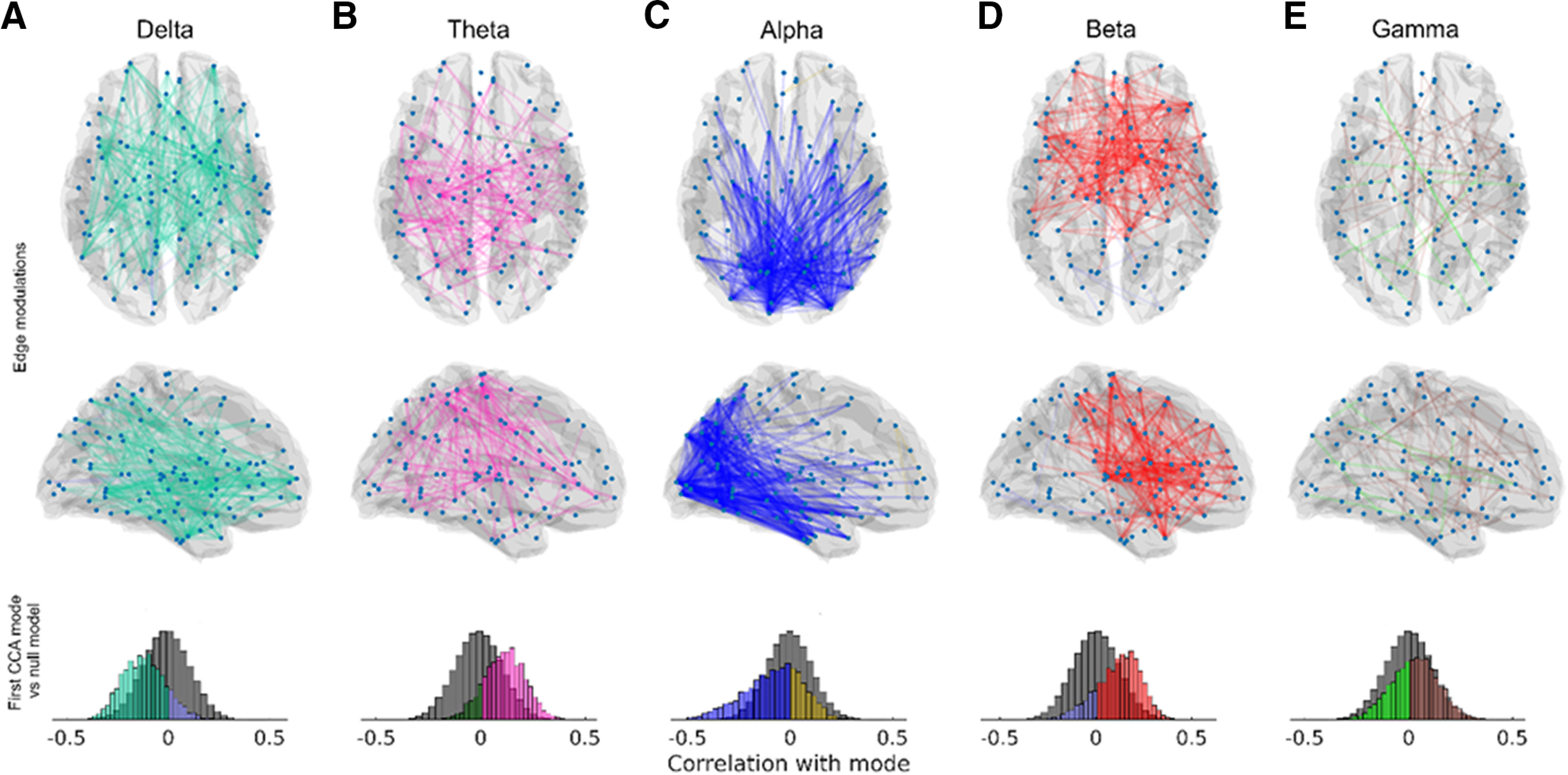
***A–E***, MCC (i.e., edges whose connectivity on a subject-by-subject level covaries with the mode, and thus behavior, in short MCC) for each frequency band, from δ-band, θ-band, α-band, and β-band to γ-band. MCC values of frequency bands are color coded (two colors per band, positive and negative, encoding the correlation coefficients, in a range from –0.5 to.5 to the observed canonical mode). Edges rendered on the brain templates are thresholded at the 0.5th bottom and 99.5th top percentile (of the permutation-based null distribution) for visualization. Each of the bands shows a preference for either positive or negative relationship to the mode but not a mixture of both. This is also visible in the histograms in the bottom row that depict the distributions of all (i.e., unthresholded) correlation coefficients, with comparison to null distributions generated by a permutation test (*n* = 10,000, in gray). For additional control analyses demonstrating the robustness of results, see also Extended Data Figure 2-1.

10.1523/ENEURO.0101-20.2020.f1-2 Extended Data Figure 1-2 Table showing the parcel identities with anatomical labeling derived from the BNA atlas (Jiang, 2013). Mention of several areas means the parcel has been created by merging these originally separate BNA parcels into a single parcel. m = medial; l = lateral; r = rostral; a = anterior; p = posterior; v = ventral; c = caudal; i = inferior; o = orbital; p = pregenual; ag = agranular; rv = rostroventral; sg = subgenual; cv = caudoventral; cl = caudolateral; dg = dorsal granular; lv = lateroventral; dm = dorsomedial; vm = ventromedial; tla = tongue and larynx area; ms = medial superior; iv = intermediate ventral; rd = rostrodorsal; cd = caudodorsal; rp = rostroposterior; op = opercular; vla/d = ventral a/dysgranular; da = dorsal agranular; ms = medial superior; ulhf = upper limb, head, and face region; op = opercular; pg= pregenual; ms = medial superior; ll = lower limb region; dld = dorsal dysgranular; iv = intermediate ventral; pc = postcentral; ip = intraparietal; vld = ventraldysgranular; vlg = ventral granular; dla = dorsal agranular; cvl = caudal ventral lateral; ta = temporal agranular; lp = lateral posterior. Download Figure 1-2, DOCX file.

This parcellation is different to that used to analyse the relationships between fMRI connectivity maps and behaviour (Smith et al., 2015) and also offered as part of the HCP1200 release. We chose to use the above data-driven customized parcellation based on the rationale that the fMRI-derived parcellation is a spatial ICA capitalizing on the high spatial resolution available in fMRI data, which sometimes results in spatially fine-grained components. This level of spatial resolution is not always a given in MEG data (as attested by the rank deficiency of the 200 parcel model described above). For control analyses that employed fMRI-derived measures of functional connectivity we employed the previously published approach (Smith et al., 2015), but chose the lower resolution of N=100, corresponding to the dimensionality chosen for MEG analysis.

To obtain parcel-time series, the first principal component over all voxels belonging to any given parcel was extracted. After extraction, resulting parcel time-series underwent reduction of source leakage by using a multivariate leakage reduction approach (Colclough et al., 2015), effectively orthogonalising all parcel time-series and removing zero-lag correlations.

All analyses were conducted on a Linux server with a 16-core Intel(R) Xeon(R) Gold 6130 CPU at 2.10GHz and with 96GB of RAM, running Ubuntu 18.04. Analysis scripts were run using Matlab R2018b.

### Analysis of functional connectivity

After source-leakage correction, we estimated functional connectivity (FC), separately for each individual and session. Functional connectivity was estimated using the Oxford software library (OSL) toolbox (https://github.com/OHBA-analysis/osl-core). We first computed envelopes in 20 contiguous frequency bins, with a frequency range of 0.5 to 40 Hz, and a resolution of 2 Hz, using the resulting envelopes in each bin after Hilbert transformation of the narrow-band filtered time-series. Within each bin, full linear Pearson correlation was computed between all possible pairings of parcel-based envelope time-series, resulting in a 100 x 100 connectivity matrix for each resting state run, frequency bin and subject. After FC estimation, all three runs were then averaged to increase stability of FC estimations. After averaging, connectivity measures, i.e. the correlation coefficients were first Fisher z-transformed.

We observed that functional connectivity in spectrally resolved MEG data is not always independent of spectral power or frequency, but that higher spectral power is linked to higher group-average envelope correlations, with connectivity being stronger (on average) in lower frequency bands (which tend to have higher power due to the well-known 1/f behaviour of human brain power spectra). In order to avoid that our findings were not simply confounded by these effects, we removed this systematic variation by pair-wise regressing out of spectral power from connectivity measures (i.e. envelope correlation coefficients) from all possible pairings (i.e. edges). This resulted in subject-specific connectivity estimates without power-induced low-frequency bias. After this, we removed the other previously defined confounds (see Methods, “Subjects and Behavioural measures”). Subsequently, the resulting connectivity matrices were renormalized to ensure zero mean and unit variance.

For further analyses we chose to further reduce the number of frequency bins to 5 bands, so we averaged the initial spectrally resolved connectivity data to approximately follow conventional frequency band arrangement: delta-band [0.5 - 3 Hz], theta-band [3-7 Hz], alpha-band [7-13 Hz], beta-band [13-25 Hz] and lower gamma-band [25-40 Hz]. Before further analysis, we extracted the upper triangle from each symmetric connectivity matrix, concatenated the resulting matrices (5x4950x89) frequency bands (yielding a 24750 x 89 matrix FC1).

### Dimension reduction and analysis of brain-behaviour mode by means of canonical correlation analysis (CCA)

Behavioural as well as connectivity data were subjected to principal component analysis (PCA). PCA is a popular choice for dimension reduction and pre-whitening of input into linear regression models in general, but also a popular choice of dimension reduction in the specific context of a subsequent canonical correlation analysis (Correa et al., 2008, 2010a,b; Smith et al., 2015; Tsatsishvili et al., 2015; Yang et al., 2019). For connectivity, PCA is performed on the concatenated connectivity data sets (i.e. on the 24750 x 89 concatenated connectivity matrix (FC1), see Extended data Figure 1-3 for visualised approach), for behaviour it is performed on the 131 x 89 behavioural data set. Aiming for a ratio of 1 to 4 regarding variables vs observations, for both data sets, we retained 22 components per subject (22 PCs or latent variables, vs 89 subjects or observations, thus reducing the chance of overfitting (see also Methods “Validation” section for more details) in downstream analyses. This ratio, while being rather conservative, still ensured sufficient detail retained in the reduced model, meaning that after reduction to 22 components, 74.1% and 64.5% of the total variance was still explained for the behavioural data and the connectivity data, respectively. This resulted in a 22 x 89 PCA-reduced connectivity matrix, FC2 and a 22 x 89 PCA-reduced behavioural matrix (B2).

**Figure 3.**
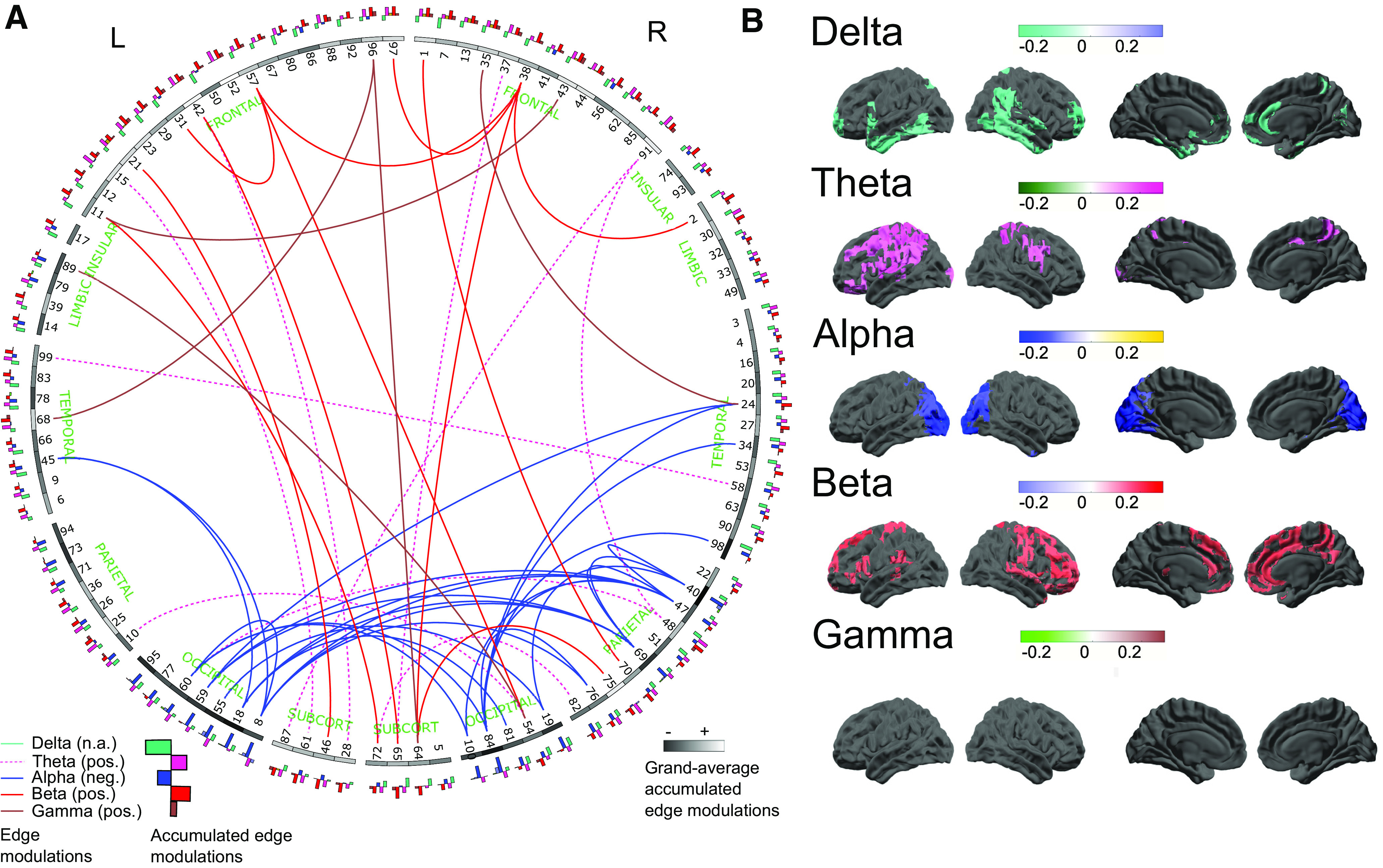
***A***, Connectogram-style visualization (Irimia et al., 2012) of the MCC edges presented in Figure 2. This figure shows the top 50 MCC edges (i.e., the top 50 positive and negative edges, respectively), across all five frequency bands (globally tresholded across all bands pooled). Nodes are represented on the outside of the ring, in an approximately anatomically-faithful anterior-posterior and left-right arrangement. Anatomically labeled parcel identities are listed in Extended Data Figure 1-2. Each parcel’s grayscale value indicates its average relationship to the CCA mode, i.e., its average accumulated MCC values across frequency bands, white indicates a positive relationship to the mode, and black indicates a negative relationship (averaged over bands and all edges, i.e., connections of that parcel). The five-band histograms on the outer ring indicate the band-specific accumulated MCC values for each node (same color coding as for the top edges in Fig. 1*A* and the maps in Fig. 1*B*, histograms pointing outwards are positive, inward histograms indicate negative accumulated MCC values). ***B***, Maps of accumulated MCC values for all frequency bands. These represent the average MCC values, over all connections, for each parcel. Thus, these accumulated maps represent the overall involvement of each parcel in predicting behavior and provide complementary information to the MCC edges by showing the presence of connectivity foci, i.e., nodes of particular importance to the MCC network, acting as sinks or hubs. Thresholded at the 10th and 90th percentiles for negative and positive accumulated MCC values, respectively. NB, no suprathreshold accumulated MCC values are observed in the γ-band. L = left; R = right; pos. = positive; neg. = negative; SUBCORT = subcortical.

10.1523/ENEURO.0101-20.2020.f1-3 Extended Data Figure 1-3 Illustration of analysis approach. FC as defined by envelope correlations within five conventional frequency bands are extracted from *n* = 89 subjects. For each subject, these spectrally resolved connectivity features are first concatenated (A, forming matrix FC1) and subjected to group PCA, retaining the first 22 principal components per subject (resulting in matrix FC2). The same approach is used for reducing dimensionality of the subject measures of all subjects (concatenated vector B1), retaining the first 22 principal components (resulting matrix B2). These two matrices (FC2 and B2) are then subject to CCA, which identifies within each matrix the optimal linear weighting to maximize correlation of features between the two sets of variables (i.e., brain vs behavior), transforming matrices FC2 and B2 into FC3 and B3. There, each row represents one mode where the newly formed (i.e., linearly recombined) connectivity and behavior canonical variates correlate most strongly (first mode here is indicated by thin black line). Download Figure 1-3, TIF file.

After PCA-derived dimension reduction we applied canonical correlation analysis (CCA) to the PCA-reduced data. CCA is a method that identifies the linear combination in each of two sets of features that transforms them to produce a maximal match of the two sets. The linear combinations that maximize correlation between the two sets of features or matrices are typically referred to as “modes”. We employ CCA here to examine whether there is such a match, or mode, of brain-behaviour population covariation for MEG functional connectivity and behavioural data. In contrast to fMRI approaches, MEG contains the additional dimension of frequency, here characterised in five frequency bands. CCA is then performed on these two identically-sized matrices (FC2 and B2), finding the linear combination of weights (or mode) that transforms each of the two matrices into new matrices (FC3 and B3) where each row is maximally similar to each other. Each row in FC3 and B3 corresponds to a newly formed canonical variate that is orthogonal to all other canonical variates within FC3 and B3, representing the linear weighting of connectivity (FC2) and behavioural features (B2) to best align brain and behavioural covariation across subjects. Each row with their corresponding canonical variates corresponds to a “mode”, with decreasing degree of correlation. Consequently, CCA result in a maximum of 22 canonical modes with 2x22 canonical variates that are also orthogonal within FC3 and B3. Each mode’s statistical significance, that is, the significance of the correlation between rows in FC3 and B3 is then established by permutation testing (with n = 10000), which corrects for multiple comparisons. For permutation testing, subject labels were swapped while respecting family structure in the data. For running CCA and permutation testing we used an adapted version of the scripts made available in a previous CCA study (Smith et al., 2015).

### Visualisation of post-CCA results

For characterization of the observed CCA mode(s) with respect to the underlying behavioural variables and connectivity, the resulting behavioural CCA weights (i.e. one per subject, n=89 in total) were correlated with the full, deconfounded behavioural variables (i.e. 131 x 89), and the mode-derived connectivity weights (n=89), in turn, were correlated with the full, deconfounded individual FC networks (i.e. the original 5 x 4950 x 89 connectivity matrix), yielding interpretable results, i.e. the composition of modes in terms of the correlated behavioral variables as well as the edges, of which their connectivity strength, on a subject-by-subject level, correlated with the mode, further termed “mode-by-connectivity correlations (MCC)”, or “MCC edges”, corresponding to the concept of “edge modulations"” used previously (Smith et al., 2015). For visualization of connectivity and these resulting MCC edges, we used the freely available Circos software suite (Krzywinski et al., 2009) (http://circos.ca/software/download/circos/). We also derived MCC *nodes* from the data to reflect the average functional relevance i.e. the average MCC value, of the corresponding node. This essentially collapses one dimension of the 100x100 MCC edge matrices by averaging over edge connections. For visualizing the results, the parcellation functionality of OSL was used.

### Validation of CCA results

CCA is highly efficient in maximizing correlation between data sets, finding the features (or a linear combination thereof) that are maximally aligned. However, this naturally leads to concerns of overfitting. While it already has been shown that similar approaches have yielded robust and stable results for larger data sets (with a similar parametrization of CCA input regarding the ratio of features to sample size), the current dataset is smaller. Thus, while we are confident that our choices, e.g. a low number of retained components, a relatively modest number of parcels etc. are conservative and ensure robustness, we performed several additional control analyses to rule out overfitting and to add confidence in the stability of the presented results. The following analyses were performed:

1. In a first cross-validation test, we split each subject’s MEG resting state data set into two parts: One part consisted of the first two resting state sessions, the other part consisted of the last session. CCA was applied to the first, acting as a training set - identifying the best matching mode in the first two sessions - whereas the second data set (the remaining resting state session) acted as test set - by applying the identified weights from the training set on the test set, effectively predicting subject and connectome weights for the test set. These predicted subject measure and connectome variates for the test subjects were then correlated with each other to obtain an estimate of how well the weights identified by the CCA on the training set still work in the test set. Mean correlation in the first mode, i.e. between the first canonical variate of subject measures and the corresponding first canonical variate of connectome data in the training set (FC3_(1)train_ vs B3_(1)train_) was 0.9X, while the brain-behaviour correlation coefficient in the test set (FC3_(1)test_ vs B3_(1)test_) was at 0.79. As a reference, the mean correlation of the permutation null distribution in the test data set was 0.02, with a standard deviation of 0.11. At the observed correlation of r=0.79, significance of the observed correlation was at max p = 1x10^−4^ (the threshold for r at a 5% alpha-level (corrected) being r = 0.21).
2. For a second, complementary cross-validation test, we used a leave-one-subject-out approach to test whether there is overfitting on a cross-subject level. Accordingly, CCA was applied to a first training set of all subjects apart from the left-out subject (n = 88) - identifying the best matching mode in these subjects - and applying these weights (that form the first canonical variates) on the left-out subject set effectively obtaining predicted subject and connectome canonical variates for the left-out, test subject. Subsequently, this approach was repeated for all 89 subjects, resulting predicted subject measure and connectome scores for all subjects. These predicted subject measure and connectome scores for the test subjects were then correlated and tested for significance. For this type of cross-validation, correlation coefficient r in the first mode, i.e. between the first canonical variate of subject measures and the corresponding first canonical variate of connectome data in the training set (FC3_(1)train_ vs B3_(1)train_) was at r = 0.67. Mean correlation coefficient r of the permutation null distribution was <0.01, with a standard deviation of 0.11. Significance of the correlation of the first mode was at maximum of p = 0.001 (with a 5% significance threshold of a correlation coefficient r being 0.1930).
3. In this control analysis, we tested the stability of our spectrally resolved approach with regard to the resulting band-specific MCC edges, i.e. the functionally relevant networks identified in each of the five bands. To do so, we now split the connectivity data, for each subject, into 5 sets, each containing only a single frequency-band specific connectivity matrix and then ran the described PCA-CCA pipeline separately on those. We then compared the resulting separate MCC matrices (MCC_sep_) with the corresponding band-specific MCC matrices of the original, integral CCA analysis (MCC_orig_), where all 5 bands entered CCA in their entirety (we chose this correlation as a test criterion here, since in this case neither canonical subject measure variates nor functional connectivity variates (FC3, B3) are directly comparable to the one integral main analysis. The resulting correlation coefficients between MCC_sep_ and MCC_orig_ were: in delta band 0.76, theta band 0.48, alpha band 0.80, beta band 0.48, and in the gamma band 0.32. For interpretation of these results it needs to be said that a holistic, integral CCA of all five band-specific connectomes is not expected to give identical results in each band as the band-separate analysis in the control condition, but it answers the question whether the general relevance, for example the quality of each identified band-specific network is preserved (see the “CP positive and negative” section in Discussion). All bands show, on average, the same qualitative relevance – being predominantly either CP positive or negative, as in the original analysis. For an exemplary illustration of the alpha-band MCC edges in analysis 2 and 3 see Extended data Figure 2-1.

For the remaining control analyses we will present the validation and control analysis results in the following order: CC1, CC2 and CC3; with CC1 being the correlation coefficient between the first canonical variates, representing the first canonical mode in FC3_orig_ and FC3_control_, and CC2 being the correlation between first canonical variates in B3_orig_ and B3_control_, while CC3 is the resulting brain-behaviour correlation (FC3_alternative_ vs B3_alternative_) in the first canonical mode:

1. In this control analysis, we ran a CCA on the same 89 subjects, where we only used fMRI derived connectivity measures, analogous to previous reported approaches. Similar to our main result and previously reported fMRI results, we found one significant mode arraying subjects on a positive-negative axis of behavioural variables. Resulting connectome (FC3_MEG(1)_) and subject measure scores (B3_MEG(1)_) for the first canonical mode of the MEG derived main analysis (5-bands), and the fMRI derived canonical scores (FC3_FMRI(1)_ and B3_FMRI(1)_ for the first mode correlated by CC1 = 0.26 and CC2 = 0.27, respectively. When comparing these fMRI derived CCA results against the full 20-frequency-bin MEG-CCA correlations were slightly higher: CC1 = 0.3 and CC2 = 0.32, respectively. While these correlations seem relatively low, qualitatively, the fMRI-derived CCA approach identified a positive-negative axis within the first canonical mode similarly to previously reported and identified here by the main MEG CCA analysis, see Extended data Figure 1-4 for the behavioural variables associated with and their ranking within this mode. The resulting first canonical mode yielded a correlation coefficient of CC3 = 0.92.
2. We compared the CCA outcome of our 5-band approach to the outcome using the initially estimated, ‘fully’ spectrally resolved 20-bin connectivity data (see Methods above). Correlations of subject measure scores and connectome scores (i.e. FC_orig_ vs FC3_control_ and B3_orig_ vs B3_control_) were at CC1 = 0.8 and CC2 = 0.85, respectively. The resulting mode gave a brain-behavior correlation coefficient of CC3 = 0.93.
3. In this control analysis we did not perform source leakage. Correlations between the original, full-source leakage corrected model and the non-corrected model were: CC1 = 0.25 and CC2 = 0.27 for the first canonical variates (FC3_orig_ vs FC3_control_ and B3_orig_ vs B3_control_). Resulting brain-behaviour correlation was CC3 = 0.89.
4. A model where we only used 50 parcels (fMRI-derived parcellations from the HCP initiative, HCP1200 release available from https://db.humanconnectome.org/data/projects/HCP_1200) for parcellation of MEG data instead of the 100 in our original analysis, correlation to the original canonical variates were CC1 = 0.14 and CC2 = 0.16 respectively (FC3_orig_ vs FC3_control_, B3_orig_ vs B3_control_). Resulting brain-behaviour correlation was CC3 = 0.90.
5. In another control analysis, we chose an approach where connectivity was defined as broadband connectivity (1-40Hz) only, so instead of several bands or bins there was only one connectivity measure per parcel. Correlations with the original canonical variates (FC3_orig_ vs FC3_control_ and B3_orig_ vs B3_control_) were CC1 = 0.36 and CC2 = 0.38. Resulting brain-behaviour correlation was CC3 = 0.90. The correlation coefficient of each identified first mode for control analyses 4-8 can be found visualized in Extended data Figure 1-5*A*.
6. In a last control analysis, we tested whether MEG and fMRI connectivity show some overlap in explaining variance across the subject population, i.e. we tested the link between these two independently acquired and modality-specific connectomes. This test was performed by using only connectivity data as input to the CCA, i.e. one set of variables being the fMRI connectivity data while the other one was the MEG based connectivity data (from the same subjects), asking for the existence of a connectome-connectome mode of population covariation. As result, we observed one significant mode, tying together MEG and fMRI connectivity across subjects was identified (resulting brain-behaviour correlation coefficient in first mode was r = 0.92). Apart from the demonstration of the existence of such a link, we did not perform further analyses to look into spatial or spectral components of this link or visualize them, since it was beyond the scope of this study.

10.1523/ENEURO.0101-20.2020.f1-4 Extended Data Figure 1-4 First CCA mode using fMRI-derived connectivity (*n* = 89). ***A***, Behavioral ranking (thresholded at |rho| > 0.25) and brain-behavior mode visualization across subjects. ***B***, Correlation of CCA-derived subject measure scores and connectivity scores for the first canonical variates identified, i.e., the first canonical mode. In color, the behavioral score for the top-ranking behavioral variable in this mode, here line orientation discrimination, is shown per subject. Download Figure 1-4, EPS file.

10.1523/ENEURO.0101-20.2020.f1-5 Extended Data Figure 1-5 Comparison of different CCA analyses. ***A***, Correlation of first canonical variates (first mode) of connectivity versus behavior (*y*-axis show level of significance of correlation in negative logarithmic scale). ***B***, Explained variance (in percent) of the behavioral variables in the first mode, same connectivity models as in ***A***. In the order of appearance: (1) original analysis (five frequency bands, 100 parcels); (2) 20-bin resolved MEG connectivity data; (3) fMRI connectivity used instead of MEG connectivity; (4) spectrally unresolved MEG broadband connectivity (1–40 Hz) used; (5) 50 parcel MEG connectivity data used; (6) as original analysis, without source leakage correction performed. See also Materials and Methods, Validation of CCA results (analyses numbered 4–8). Download Figure 1-5, EPS file.

We also computed the explained variance of the first and all following canonical variates, i.e. the first and significant CCA mode until the last computed mode with regard to the set of behavioural variables. Results are shown in Extended data Figure 1-6, demonstrating that the first canonical mode explains significantly more variance in behaviour than the permutation based null model where we swapped subject label n=1000 times. For a comparison of the explained variance in the control analyses 4-8, see also Extended data Figure 1-5*B*.

10.1523/ENEURO.0101-20.2020.f1-6 Extended Data Figure 1-6 The amount of variance (%) explained by the first canonical variate (i.e., first mode) in the full set of behavioral variables. Permutation test *n* = 1000. Green and red lines indicate differently thresholded top and bottom percentiles of permutation based null model. The first mode explains significantly more variance than permutation-based (pseudo-)results. Download Figure 1-6, EPS file.

10.1523/ENEURO.0101-20.2020.f2-1 Extended Data Figure 2-1 Stability of MCC results for different analyses ***A***, Exemplarily for the α-band, MCCs for the original (main) analysis, and three control analyses. First panel, Results from original analysis, visualizing the α-band MCCs. Second panel, CCA has now been performed separately for each frequency band, here the MCC result for the α-band model is shown. Third panel, For this analysis, data were split into a training and test set, where the training was carried out on the first two resting-state sessions and testing was carried out on the third session. α-Band MCCs are shown for the CCA mode that was learned in the training set and applied to the test set. Fourth panel, In this analysis, data were split into training and test set by a leave-one-subject-out approach. α-Band MCCs are shown for the CCA mode as established for the left-out subjects (and their predicted canonical variates; for details, see Materials and Methods). All α-band MCCs show comparable patterns with the majority of suprathreshold patterns being negative, posterior MCC patterns related to the identified CCA mode. ***B***, Summary of the distribution of suprathreshold MCCs across all frequency bands. Complementary to first row, this shows all frequency bands from δ to γ. Almost all analyses behave the same way, apart from the β-band in the frequency band separate CCA analyses (where β-band CCA did not yield any significant mode). ***C***, Similarity of MCC patterns to the original main analyses (first panel and Fig. 2). Here, correlation was performed over the whole 4950-element MCC vector in each frequency band. High correlations indicate good correspondence with original MCC patterns from the main analysis (as visualized in Fig. 2). See also Materials and Methods, Validation of CCA results (analyses 1–3). Download Figure 2-1, EPS file.

#### Data availability

The sensor space MEG resting state data and the corresponding subject measures are available online on https://db.humanconnectome.org, however they are not publicly accessible without registration. Due to privacy concerns, access to these data needs registration and approval by the HCP consortium. All authors have been approved and have accepted the terms of use for the open and restricted part of the HCP data.

The processed and derived data (functional connectivity, CCA results etc.) that support the findings of the study can be made available upon reasonable request to the corresponding author. Since derived from the HCP data, the processed data are not publicly available due to them containing information that could potentially comprise research participant privacy and/or violate HCP terms of use and thus will be only shared accordingly.

#### Code accessibility

The code used in this study comes from several publicly available toolboxes and software. A previous implementation of CCA with respect to HCP functional connectivity and subject measures has been used elsewhere (Smith et al., 2015) and can be found on https://www.fmrib.ox.ac.uk/datasets/HCP-CCA/. Circos software is available on http://circos.ca/software/download/circos/. OSL software is available on https://github.com/OHBA-analysis/osl-core. Customized Matlab scripts for source reconstruction, use of PCA/CCA, post-CCA visualizations using OSL and Circos will be made available upon reasonable request to the corresponding author.

## Results

### MEG-derived functional connectivity reveals a global brain-behaviour mode along a positive-negative axis of subject measures

We used canonical correlation analysis (CCA) to examine the relationship between spectrally resolved resting-state MEG connectivity and a battery of 131 behavioural, demographic and personality variables in 89 healthy young individuals. CCA is a method that identifies an optimal linear combination of features (a mode) in two separate data sets that maximizes correlation between them – in our case functional, spectrally resolved MEG connectivity and subject measures. MEG connectivity was examined in five conventional frequency bands in the range 1-40Hz (delta [0.5 - 3 Hz], theta [3-7 Hz], alpha [7-13 Hz], beta [13-25 Hz] and lower gamma [25-40 Hz]). Connectivity was determined for the whole brain divided into 100 functionally-defined parcels (see Extended data Figure 1-1 and Extended data Figure 1-2).To remove simple effects of resting-state power, average resting-state spectral power was regressed out of connectivity strength variations across subjects in a pairwise manner, i.e., for each connection, and frequency band, power of both nodes was regressed out. CCA was then performed on a PCA reduced subspace of this data, examining the potential link between subject-wise variations of connectivity strength and behavioral performance (see Materials and Methods), identifying one significant canonical mode after permutation testing (*r* = 0.94, *p* < 10^−4^; for a visualization of the CCA approach, see also Extended Data Fig. 1-3). This mode links spectrally resolved brain connectivity and behavior. The existence of a significant mode indicates that there is a significant relationship between resting-state connectivity and the subject measures. This first canonical mode is also unique in that it explains more variability in behavior than the other modes (or permutation-based null models), as shown in Extended Data Figure 1–6.

In order to provide a functional characterization of this mode of maximum covariation between subject measures and connectivity, we evaluated how the mode (i.e., the observed first canonical variates) covaries with the individual subjects’ scores on each of the subject measures and connectivity measures. This procedure reveals the way in which the mode that best aligns the full set of subject measures with the connectivity patterns explains the interindividual variation in each of the subject measures. Thus, positive correlation between a subject measure (SM) and the canonical variate suggests that the higher a subject is ranked on this subject measure, the larger this subject’s score for the canonical variate. Conversely, a negative relationship between an SM and the canonical variate indicates that higher scores on that SM are associated with lower subject scores on the canonical variate.

The behavioral variables most strongly positively and negatively associated with this mode are shown in Figure 1*A*. The subject measures are arranged along a positive-negative axis that is qualitatively the same as that previously described for an fMRI-derived CCA mode (Smith et al., 2015). It ranges from subject measures that entail tobacco consumption and somatic problems (which may quite reasonably be characterized as “negative”) to subject measures indicating high cognitive performance (CP; at the other end of this ranking, interpreted as “positive”). The nature of the positive relationship between the mode and performance on a working memory task is further illustrated in Figure 1*B*, where a clear pattern emerges demonstrating that higher individual scores on the subject measure and connectome canonical variate are predictive of superior list sorting performance.

This outcome alone is remarkable, despite using a different imaging modality and having access to only a substantially smaller sample (89 vs 461 datasets), the nature of the mode revealed is highly consistent with that previously demonstrated for the same set of subject measures and rs-fMRI connectivity. A quantitative comparison with rs-fMRI-based CCA results, as well as the impact of parcellation scheme and subdivision into the selected frequency bands on the outcome, is presented in Extended Data Figure 1–5.

Although this MEG-derived canonical mode resembles and partially even reproduces a previously identified fMRI-derived brain-behavior mode, we are at pains to point out that this does not automatically render it the “universal” brain-behavior mode. Given a different array of subject metrics, or a qualitatively different array of brain metrics, the principal brain-behavior mode uncovered by CCA could be different to the one at hand. This possibility is, however, speculative. What we may state with confidence is that, independent of the underlying imaging modality, a canonical brain-behavior mode, optimally matching spectrally resolved connectomes and subject measure, can be identified, which arranges subjects on a positive-negative axis consistent with a study in which connectivity was derived from hemodynamic measures of brain activity.

### The CCA mode is composed of spatio-spectrally segregated subcomponents

In a second step, we characterize the connectivity components of the identified brain-behavior mode, for each of the five frequency bands and their connectomes. In order to do this, the connectivity patterns across subjects in each band were correlated with the individual connectome weights of the significant CCA mode. This reveals which connections (hereafter referred to as edges) of the connectome are more, or less, related to the mode, and therefore associated with individual differences over subject measures. The outcome of this analysis is shown in Figure 2. The correlation coefficients calculated in this analysis are referred to as MCC or MCC edges. The patterns of within-band MCC edges significantly related to the mode are clearly differently distributed over the brain and have distinctly negative or positive relationships with the mode in each of the analyzed frequency bands.

Figure 3*A* shows the top 50 MCC edges across all five frequency bands, providing an overview of the diversity of connectivity patterns in each of the frequency ranges analyzed. To reveal the relative functional importance of each node in terms of its relationship to the mode, averaging was performed over all the MCC correlation matrices in which the respective node is involved. We thus derive maps of accumulated MCC nodes, which are shown in Figure 3*B*. As described above, the distributions of these accumulated MCC maps across bands is primarily unipolar, each of the five frequency bands preferentially showing either a positive or a negative relationship with the mode, but not both (Fig. 3*B*).

### Connectivity of spectrally-resolved brain activity is preferentially positively or negatively linked to CP but not mixed

The discovery of frequency-specific connectivity components that correlate either negatively or positively with the CCA-derived mode, and as a consequence, negatively or positively with CP, invites the conclusion that some connectivity patterns are positive, while others are negative. We will refer to them further as CP positive and CP negative. This interpretation is somewhat consistent with previous reports from rs-fMRI data (Smith et al., 2015), which also showed that certain subcomponents (especially areas pertaining to the default mode network) of the whole-brain connectome are positively, and others (mostly visual areas) negatively, associated with the positive-negative axis behavioral exposed by CCA.

#### δ-Band and α-band MCC show negative relation to CP

α-Band and δ-band connectivity were almost exclusively negatively associated with the CCA mode, i.e., negatively correlated with CP. The parcels with the highest incidence of significant connectivity-behavior relationships in the α-band are principally concentrated in visual-posterior areas, consistent with the usual distribution of α power at rest. Given that spectral power was regressed out of these analyses (see Materials and Methods), we may conclude that higher connectivity in the α-band, absent the biasing effects of the typically higher power in this frequency range compared with the rest of the spectrum, is predictive of lower CP and higher incidence of negatively associated health and behavior indicators. δ-Band connections also correlate negatively with behavior. The majority of implicated nodes are differently distributed compared with α-band MCCs, incorporating bilateral inferior posterior and anterior temporal lobes and inferior and orbitofrontal areas.

The negative relationship of the α-band posterior MCC edges to the canonical behavioral mode is comparable to the accumulated MCC nodes known from fMRI-derived CCA analysis (Smith et al., 2015). By means of spectrally resolved CCA analysis, we can assign a neuro-spectral profile to some of these previously reported MCC networks. For example, the fMRI-derived negative-posterior MCC nodes in early visual areas (Smith et al., 2015) are putative equivalents of the MEG-derived negative-posterior α-band MCC nodes, exhibiting similar locus and functional relevance (i.e., higher connectivity = lower score in a similar CCA mode).

The components of the δ-band are less easy to map onto previous results. The finding of δ-band-specific MCC values negatively related to the mode is suggestive of a negative impact of increased low-frequency neural coupling at rest on cognitive function. Usually, pronounced δ oscillations are found during sleep, while in the awake human brain, δ is most often recorded in pathologic situations such as in the presence of lesions or tumors. Unusually elevated δ coupling may also be a sign of slowed θ oscillations, a phenomenon often observed in pathologic cognitive decline, such as Alzheimer’s disease, caused by cholinergic loss (Speckmann and Elger, 2005). While we do not propose that the young, healthy cohort examined here suffered from any neurologic pathologies, it is intriguing to note the fact that slow oscillatory activity is not typically associated with optimal brain function during wakefulness.

#### θ-band, β-band, and γ-band MCC edges show positive relationship to CP

β-band, θ-band, and γ-band MCC edges exhibit a substantially different relationship to the canonical mode, compared with α and δ MCC edges. This is the case both in terms of the polarity of the relationship, and the spatial distribution of the relevant nodes. For the observed canonical mode, subjects scoring high in frontal and prefrontal β-band connectivity also score highly in the identified canonical mode, indicating higher CP. The patterns that arise from β-band accumulated MCC maps, especially in medial areas, show good correspondence to previous results for fMRI connectivity (Smith et al., 2015). For θ MCC edges and accumulated MCC maps, spatially we see a more precentral pattern with a functionally similar interpretation, the more pronounced the connectivity is in these edges, the higher the subject scores in the canonical mode, i.e., the higher the subject is ranked on the positive-negative axis identified by the significant CCA mode. The properties of γ-band MCC edges are particularly noteworthy. While the overall γ MCC network is somewhat patchy, featuring what appear to be a greater number of short-range connections, its connectivity is still relevant for the global mode of behavior. It also seems to have fewer sinks or hubs where connections emerge or terminate, resulting in the absence of significant accumulated MCCs, in contrast to the more clustered network patterns in frequency bands at lower frequencies. Considering that γ in general is known to be an index of local activation, offering often relatively fine spatial resolution (e.g., down to mapping the representation of individual digits of the hand; Miller et al., 2009), this is not altogether surprising.

This striking dichotomy of either CP positive or CP negative connectivity, when looking at spectrally resolved connectomes, is both novel and unexpected. Previous results, using fMRI connectivity that is agnostic to the underlying frequencies of neuronal communication, have so far only shown patterns of mixed positive or negative relationship to the observed brain-behavior mode. We tested whether this segregation does arise from the CCA trivially by creating MCC edges from surrogate data, by permuting the subject-labels and recomputing the (pseudo-)MCC patterns. The resulting null-distributions of correlation coefficients are shown in Figure 2, bottom row; they do not exhibit any polarity preference or skewedness as the actual MCCs do.

### Resolved MEG connectivity model shows results comparable to fMRI connectivity

In terms of the resulting canonical mode and its maximum alignment of connectivity and subject measures, the MEG connectivity model used here (five bands, 100 parcels) shows comparable results with regard to the results obtained with fMRI connectivity (for identical subjects, subject measures and number of principal components entering CCA, see Extended Data Fig. 1-5*A*). Also, with regard to explaining the full set of subject measures, the MEG connectivity used here performs similarly well as the fMRI-based connectivity (Extended Data Fig. 1-5*B*). Interestingly, what was also observed is that an entirely unresolved, i.e., 1D broadband connectivity model performs less well in terms of maximum alignment and explained variance of subject measures. Even when using the initially available spectral resolution (20 2-Hz wide bins instead of five bands), results did not outperform the five-band model.

## Discussion

In summary, we show that exploiting the spectral richness of MEG connectivity patterns by using a spectrally resolved connectome reveals a brain-behavior mode not unlike previous results uncovered via hemodynamic connectivity metrics. Beyond simply reproducing these, our results demonstrate the existence of functionally distinct networks across a broad range of conventional frequency bands. Even neighboring bands, e.g., α and β, can have opposing functional roles with respect to a brain-behavior mode arranging behavioral measures on a positive-negative axis.

Regarding the approach taken in this study, the concept of spectrally resolved connectivity, which can also be considered neuronal communication at multiple time scales or channels, is not new. Some models of how neuronal assemblies communicate have focused on activity confined to certain frequency bands (Fries, 2005), while more recent perspectives also incorporate multichannel or multifrequency scenarios (Fries, 2015). Furthermore, empirical studies, mostly MEG, have also increasingly shown connectivity and networks at multiple frequencies or showing features or states with specific spectral features or profiles (Hipp et al., 2012; Brookes et al., 2016; Colclough et al., 2016; Vidaurre et al., 2018) and first reviews about the accumulated literature emerge (Sadaghiani and Wirsich, 2020). What was, however, hitherto missing was a functional characterization of these spectrally resolved features or parameters, specifically of connectivity, because of the absence of an attested link to behavior. Here, to the best of our knowledge, for the first time, we have demonstrated the nature of the relationship between a spectrally resolved connectivity estimate to not just one, but an entire battery of behavioral and other subject variables.

Although the classification of brain activity into several predefined frequency bands is already an extension of spectrally unresolved approaches, it still might be an oversimplification of the richness of neuronal dynamics in the brain. However, it can be useful, and the fact that using predefined, canonical, spectral ranges and the corresponding connectomes reveals a global brain-behavior mode with little overlap between bands lends support to the idea of spectrally resolved, functionally meaningful networks. The relevance of these bands is underscored firstly by the comparison with two analyses reported above. First, the analysis using broadband connectivity showed a brain-behavior mode that was both less capable of explaining variance in behavior than the spectrally resolved approach. Second, analysis using the fully (i.e., 20-bin) spectrally resolved connectome failed to reveal any additional insights into brain-behavior associations compared with of the five conventional bands. Of course, this does not mean that deploying the standard five-band model is the optimum for identifying brain-behavior links, but it suggests the existence of well-defined and functionally distinct frequency ranges and the networks that emerge from them.

Our study and its results add several novel aspects here. First, we show that an observed link between brain and behavior cannot be attributed to a single isolated frequency band but is what appears to be a rich multilevel spatio-spectral connectivity pattern. Second, the holistic analysis that we offer here is identifying a clear, interpretable, and plausible brain-behavior axis which has been reported before, and in the present case of M/EEG connectivity adds a novel component which is looking at neural communication in a spectrally resolved and spatially interpretable manner, in a healthy population. By virtue of this analysis, we were able to reveal different contributions of the spectrally resolved connectome(s), with the striking observation is that the frequency bands and their functionally relevant connectomes often can have very different functional roles, some of them having a positive functional relationship with CP and others a negative relationship.

### The multispectral character of positive and negative MEG connectivity

This strong preference for either positive or negative relationships with the mode, corroborated by numerous control analyses, seems to be supportive of the existence of different channels of neuronal communication, characteristic of and confined to these frequency bands and the edges involved. Furthermore, we do not find that a single frequency band completely dominates the CCA-derived brain-behavior mode. Rather, analyses of both the functionally relevant patterns of connectivity, the MCC edge correlations, as well as the accumulated MCC node correlations, point to a relatively distributed and balanced contribution of network components indicative of behavior.

The fundamental and qualitative difference between the observed patterns of MCCs in α-band and β-band is a good illustration of our observations regarding the positive or negative nature of spectrally resolved connectivity. While both rhythms, α and β, at rest, express FC network patterns that are relatively amenable to interpretation (Brookes et al., 2011b, 2016; Hipp et al., 2012), i.e. they resemble visual or motor related networks, the resulting MCC edges, for the observed mode at least for β, are more frontally or prefrontally localized and can be less easily assigned directly to sensorimotor functions. In addition, higher β-band connectivity is beneficial, while higher α connectivity (in the predominantly visual nodes implicated by CCA) is apparently deleterious for CP linked to the observed mode. This implies that higher connectivity strength is not always better, but its impact on the mode (and thus behavior) appears to be a function of frequency. The finding of negative MCC values in the α range may appear somewhat surprising, however, all additional control analyses we had performed support our finding of posterior, α-band-specific connectivity being detrimental to CP. With regard to existing studies that focused on α-band connectivity, most of them were examining patient populations. Of these studies, one study did, in line with our findings, report increased occipital α connectivity in schizophrenic patients compared with healthy controls (Liu et al., 2019), which is interesting because schizophrenia often involves cognitive impairment. Other studies dealing with cognitive impairment arising from Alzheimer’s disease (Stam et al., 2006; Ranasinghe et al., 2014) reported decreased α-band connectivity related to cognitive deficits, but more in frontal or unspecific regions. Thus, while there are some clinical studies that focused on α-band connectivity (or other bands), we are not aware of a healthy M/EEG cohort where a similar large battery of behavioral variables was collected. However, the study using HCP fMRI-derived connectivity found also most of their negative MCC values in early visual areas being in line with our findings (Smith et al., 2015). This, together with the observation that CCA identified a MEG-fMRI link of connectivity patterns (see Materials and Methods, Validation of CCA results), lends further support to our findings.

### Previous work, other related lines of research and concepts

The fundamental issue of CP positive and CP negative brain patterns is also related to another line of research, which has investigated how neuronal variability predicts healthy aging and a maintained high level of CP (Garrett et al., 2011, 2013). While the approach employed here emphasized connectivity rather than variability per se, i.e., of single sensors, sources or voxels, the two concepts are not entirely unrelated. For example, neuronal variability (or activity in the widest sense) is a necessary precondition for meaningful connectivity. However, this type of common variability as it is reflected in our conceptual approach of estimating M/EEG connectivity, is not a one-way road; whether this covariation or connectivity is positive or negative with regard to CP is very specific and rather dependent on the locus and the frequency band the network is operating at.

Regarding previous work from the M/EEG side, the following should be noted: there are decades of studies linking rhythms and cognitive processing and function (Klimesch, 1999; Arns et al., 2013), with almost every frequency band having been associated with cognitive processing in one way or another, from δ (Harmony, 2013), θ (Finnigan and Robertson, 2011; Vlahou et al., 2014), and α (Klimesch, 1999), over β (Lundqvist et al., 2018) up to the γ frequency range (Ward, 2003). However, these studies often focused on sensor-wise or areal spectral features such as amplitude or phase variability, hence being agnostic of the potential relevance of any longer-distance interareal communication or network activity. Although these local M/EEG oscillations are also likely to be embedded in small-scale local networks, these are unresolvable by M/EEG imaging, we therefore distinguish these from the longer-range networks that we have investigated in the foregoing analyses. Thus, while our network-based results seem in part in line with the general literature, e.g., ascribing α and θ a role in sleep and relaxation, while showing a more positive, attention or memory-related role of θ, β, and γ activity, a direct comparison is not straightforward and may not even be entirely valid. Similarly, the resulting spatial maps of previous studies, especially at the sensor level, and our results are only comparable to a certain degree. This is not to say that there is no overlap, the spatial maps (especially the accumulated MCC maps) in θ (covering medial temporal lobe areas), α (covering visual areas), and β (covering precentral motor and premotor areas) are indeed similar to previously reported RSNs (Biswal et al., 1995; Brookes et al., 2011a,b; Hipp et al., 2012). However, the networks and maps that we observe here go beyond simply describing group level resting-state connectivity patterns or a replication of those, they describe the edges and nodes that are predictive of cognitive-behavioral performance as delineated by the identified mode.

More directly-comparable, i.e., large-scale, network-based evidence for a link between rhythms and cognitive functions remains somewhat scarce compared with the large body of literature focusing on single sensors or sources. Nevertheless, such evidence is accumulating, and a more network-centric view is gaining traction (Sadaghiani and Wirsich, 2020). For example, more recently, it has been shown that networks of these rhythms and their network properties (such as centrality, degree, small-worldness, or path length) can link spontaneous M/EEG rhythms to CP, both in healthy subjects (Langer et al., 2012; Vriend et al., 2020; Zakharov et al., 2020) or in patient populations predicting cognitive decline (Stam et al., 2006, 2007; Tewarie et al., 2012; Ranasinghe et al., 2014; Fraga González et al., 2018; Liu et al., 2019) or surgery-related improvement (van Dellen et al., 2012). While offering a network view on the functional relevance of M/EEG rhythms, these studies did not employ an integrated, spectrally resolved connectivity approach as we have.

The apparent existence of multiple, functionally-relevant frequencies opens up the possibility of multiplexing, which is an elegant concept of how communication and information transfer may be realized in the human brain (Akam and Kullmann, 2014; Fontolan et al., 2014). While we cannot completely exclude such a scenario, our results do not support this scenario. Rather, we obtained evidence for spectrally distinct networks, that are also spatially distinct in their network characteristics. The distinct character of these behaviorally relevant components may not necessarily require multiplexed communication. This highly complementary nature suggests that the idea of frequency bands is not just an entirely artificial construct but seems to delineate different functional networks. It is important to note that the networks that are reflected by the identified MCC edges here are different from pure RSNs that show coherent behavior and as such are considered connected. The networks that are reported here are rather characterized by their similar functional meaning with respect to the identified brain-behavior mode. The patterns we find, i.e., the spectrally resolved MCC edges implicated in the canonical brain-behavior mode, are for the most part spatially distinct patterns and do not show strong overlap across spectral boundaries.

### Limitations and outlook

As with any study, our study has some potential limitations that are important to keep in mind. First of all, while our study is a first step into an examination of the functional role of spectrally resolved neuronal large-scale communication, our approach is far from providing an exhaustive picture. Two notable components missing in this picture are the following. First, directionality is absent in the characterization of connectivity here, because we determine connectivity (and its strength) by looking at envelope correlations. While this is a common approach demonstrated to be useful in numerous scenarios, one apparent extension would be adding directionality or, in a stronger sense, causality, e.g., by using Granger causality or related approaches. Second, another aspect of our approach is that at the core of our analysis, while integrating all bands into one CCA analysis, connectivity is derived from single, isolated frequency bands. This prevents direct insight into the potential of cross-frequency communication or interaction for explaining behavioral variability. In fact, one could go even further and claim that all oscillatory activity is only one part of the broader picture of neuronal dynamics and including 1/f dynamics is a crucial extension of describing neuronal dynamics (and possibly, interaction). As a last important thought, it has to be emphasized that while our observation of a brain-behavior mode (with a clear positive-negative axis related to CP) and its similarity to a previous fMRI-derived brain-behavior mode (Smith et al., 2015) is reassuring, this does by no means imply that it is the only possible mode or that there can be only one (and not several). Future studies with different imaging modalities, even more subjects or additional behavioral and subject measures may elucidate the answer to this question.

## Conclusion

In conclusion, we have provided evidence that electrophysiologically-derived and spectrally resolved connectivity present in MEG resting-state data can be used to index the ranking of individuals across a large range of subject measures. It exhibits highly structured patterns that are functionally relevant and provides sensitivity comparable to rs-fMRI-derived networks in explaining variability of subject measures across a wide range of different features and domains. Furthermore, the additional dimension afforded by spectrally resolving connectivity measures opens up new avenues into a better and more holistic understanding of the roles and contributions of brain rhythms at rest that ultimately will help cast light on the intrinsic features of the brain that determine positive and negative cognitive and behavioral traits. Given recent breakthroughs in using frequency-tuned transcranial electrical stimulation techniques to enhance cognition (Reinhart and Nguyen, 2019), outlining the roles of spectrally resolved connectivity networks is all the more timely, and provides a basis for considering potential network-level targets for novel neuromodulatory interventions.

## Synthesis

Reviewing Editor: Ifat Levy, Yale University School of Medicine Decisions are customarily a result of the Reviewing Editor and the peer reviewers coming together and discussing their recommendations until a consensus is reached. When revisions are invited, a fact-based synthesis statement explaining their decision and outlining what is needed to prepare a revision will be listed below. The following reviewer(s) agreed to reveal their identity: Dustin Scheinost, Galit Fuhrmann Alpert.

This paper extends an existing analysis approach for functionally related connectivity, previously implemented on fMRI BOLD data, to MEG. The analysis is performed on whole-brain resting-state MEG data, and consists of taking connectivity data and a wide range of behavioral variables and submitting these data to a canonical correlation analysis (CCA) to find latent factors of the MEG data that are maximally correlated with latent factors of the behavioral data. As this approach is performed with MEG data, the authors were able to test different frequency bands (specifically delta, theta, alpha, beta and low gamma- up to 40z) and observed different patterns of brain connectivity across the bands.

The paper is very well written and very thorough in terms of methodology. The results support the notion of functionally distinct networks at different frequency bands, although this by itself is not a novel notion. The main results seem to be reproducing previous reported results of the methodology applied to fMRI resting state data. Also, it is an interesting concept that in the absence of any task, individual differences in the expression of five spatio-spectrally distinct resting-state networks predict a diverse array of behavioral measures, from tobacco consumption to cognitive performance. The approach could potentially be generally applied to new sets of data. However, several analysis choices should be better justified and explained. In addition, some manually tuned steps need to be pipelined for a general use.

Major comments:

- There are several steps that require choices of parameters (like the number of parcels in use, and number of principal components used to represent the data), that may form an obstacle in offering the approach as a general approach for analysis.

- Can the authors elaborate on the choice to use principal component analysis first? Is this because the original dimensions were considered too large for a canonical correlation analysis? Did the authors test this? And if so, did they try a variation of CCA to deal with the large dimensions (like a L2 penalized version or a spare CCA)? It seems less straightforward to use too related dimensionality reduction techniques in a row (PCA is just a simplification of CCA).

- How sensitive would the results be to the exact source localization procedure? Can the authors elaborate on that?

- Five conventional frequency bands were considered in the Results. Is there a reason for not applying this to high gamma (> 40Hz)? and if it was applied, why are the results not reported?

If the main purpose of switching from fMRI to MEG is temporal resolution, it would be an opportunity to look at high frequency data.

- The optimal number of parcels set to 100, with a rationale that N=200 is too high, is not entirely justified.

- The choice of 22 PCs for the representation of both subject and connectivity data is not entirely justified. Why exactly 22? And how in general should this choice be made? (“sufficient detail”?).

- The associations between specific frequency bands and specific functions are not in themselves novel. Alpha and delta waves are associated with sleep and relaxation, whereas gamma, beta, and theta are related to alertness, learning, and memory functions (see for example Klimesch (1999), ‘EEG alpha and theta oscillations reflect cognitive and memory performance: a review and analysis, Brain Research Reviews 29: 169-195; Arns et al (2013), ‘A Decade of EEG Theta/Beta Ratio Research in ADHD: A Meta-Analysis’, Journal of Attention Disorders 17:374-383). Also their network spatial distribution is in line with existing literature. This should be presented and discussed in the discussion section.

- The authors should add a detailed limitations paragraph near the end of the paper. It would help the readers understand where potential next directions are warranted and allow the authors to discuss how these limitations may affect or not affect the interpretations of their results.

- The term “edge modulations” is confusing. Can the authors not simply write out edges from the CCA or something similar? Additionally, the term is only defined in the figure caption of figure 2 and not in the main text. Finally, if the authors do decide to retain that term, the abbreviation of EMs should be removed in favor of edge modulations.

Minor comments

- Introduction: In 4th paragraph, when introducing neural oscillations, classical references from EEG/MEG are more relevant than fMRI papers and should be cited.

- Consider rephrasing the term ‘good’ or ‘bad’ brain activity/connectivity to something like supporting or interfering with cognitive performance.

- Discussion: Missing literature review of brain waves in relation to cognitive performance at the 5 analyzed bands (delta/theta/alpha/beta/gamma), along with their spatial distributions (and compared to that found in the current study).

- As with other abbreviations, the authors should spell out MEG as Magnetoencephalography the first time they use it.

- There are a few randomly capitalized words throughout the paper, which is a bit distracting.
