# Supplementary figures and images for: Resolving the Connectome, Spectrally-Specific Functional Connectivity Networks and Their Distinct Contributions to Behavior
Source: eNeuro. 2020 Sep 9;7(5):ENEURO.0101-20.2020. doi: 10.1523/ENEURO.0101-20.2020 (PMC7484267; doi:10.1523/ENEURO.0101-20.2020)

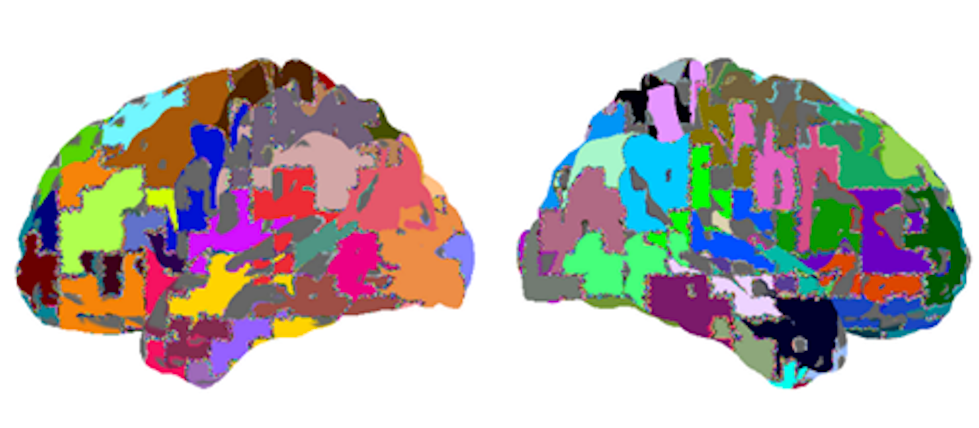

Supplement: Extended Data Figure 1-1 — Visualization of the used parcellation (n = 100, lateral view). Parcel identities can be found in Extended Data Figure 1-2, with anatomical labels. Download Figure 1-1, TIF file. [file enu-eN-NWR-0101-20-s02.tif]

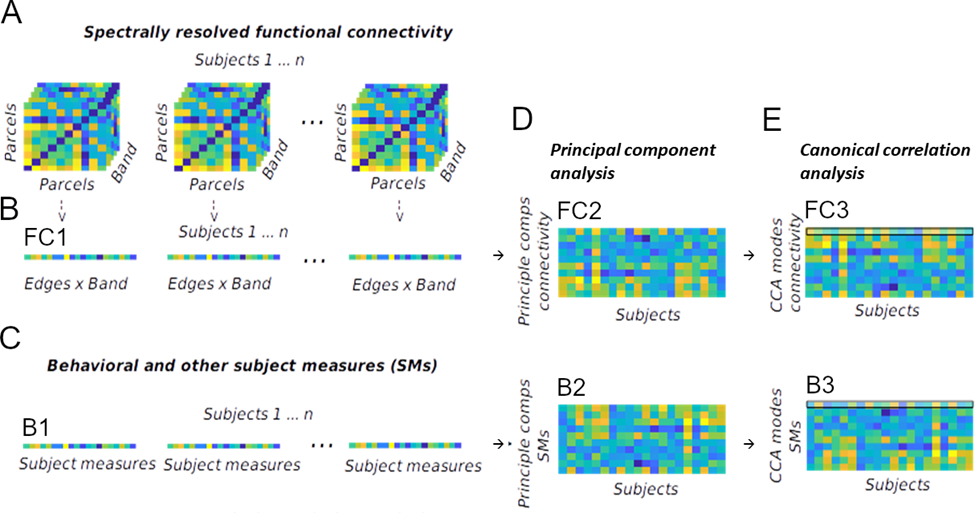

Supplement: Extended Data Figure 1-3 — Illustration of analysis approach. FC as defined by envelope correlations within five conventional frequency bands are extracted from n = 89 subjects. For each subject, these spectrally resolved connectivity features are first concatenated (A, forming matrix FC1) and subjected to group PCA, retaining the first 22 principal components per subject (resulting in matrix FC2). The same approach is used for reducing dimensionality of the subject measures of all subjects (concatenated vector B1), retaining the first 22 principal components (resulting matrix B2). These two matrices (FC2 and B2) are then subject to CCA, which identifies within each matrix the optimal linear weighting to maximize correlation of features between the two sets of variables (i.e., brain vs behavior), transforming matrices FC2 and B2 into FC3 and B3. There, each row represents one mode where the newly formed (i.e., linearly recombined) connectivity and behavior canonical variates correlate most strongly (first mode here is indicated by thin black line). Download Figure 1-3, TIF file. [file enu-eN-NWR-0101-20-s04.tif]
