# Supplementary material for: Resolving the Connectome, Spectrally-Specific Functional Connectivity Networks and Their Distinct Contributions to Behavior
Source: eNeuro. 2020 Sep 9;7(5):ENEURO.0101-20.2020. doi: 10.1523/ENEURO.0101-20.2020 (PMC7484267; doi:10.1523/ENEURO.0101-20.2020)
Supplement: Extended Data Figure 1-2 — Table showing the parcel identities with anatomical labeling derived from the BNA atlas (Jiang, 2013). Mention of several areas means the parcel has been created by merging these originally separate BNA parcels into a single parcel. m = medial; l = lateral; r = rostral; a = anterior; p = posterior; v = ventral; c = caudal; i = inferior; o = orbital; p = pregenual; ag = agranular; rv = rostroventral; sg = subgenual; cv = caudoventral; cl = caudolateral; dg = dorsal granular; lv = lateroventral; dm = dorsomedial; vm = ventromedial; tla = tongue and larynx area; ms = medial superior; iv = intermediate ventral; rd = rostrodorsal; cd = caudodorsal; rp = rostroposterior; op = opercular; vla/d = ventral a/dysgranular; da = dorsal agranular; ms = medial superior; ulhf = upper limb, head, and face region; op = opercular; pg= pregenual; ms = medial superior; ll = lower limb region; dld = dorsal dysgranular; iv = intermediate ventral; pc = postcentral; ip = intraparietal; vld = ventraldysgranular; vlg = ventral granular; dla = dorsal agranular; cvl = caudal ventral lateral; ta = temporal agranular; lp = lateral posterior. Download Figure 1-2, DOCX file. [file enu-eN-NWR-0101-20-s03.docx]

| 1 | Right Orbital Gyrus BA14 (m) | 21 | Left Middle Frontal Gyrus, BA9/46 (d) Left Orbital Gyrus BA12/47 (o) Left Orbital Gyrus, BA11 (l)  Left Orbital Gyrus BA12/47 (l) | 41 | Right Orbital Gyrus, BA13 Right Basal Ganglia, nucleus accumbens | 61 | Left occipital thalamus Left caudaltemporal thalamus | 81 | Right Medioventral Occipital Cortex, lingualgyrus (r) |
| --- | --- | --- | --- | --- | --- | --- | --- | --- | --- |
| 2 | Right Cingulate Gyrus BA24 (rv) Right Cingulate Gyrus BA32 (p) Right Cingulate Gyrus BA32 (sg) | 22 | Right Inferior Parietal Lobule BA40 (rd)  Right Postcentral Gyrus BA2 | 42 | Left Superior Frontal Gyrus, BA6 (dl) Left Middle Frontal Gyrus, inferiorfrontal junction Left Middle Frontal Gyrus, BA8 (vl) Left Middle Frontal Gyrus BA6 (vl) | 62 | Right Precentral Gyrus BA4hf  Right Postcentral Gyrus BA1/2/3 (ulhf) | 82 | Right Precuneus BA7 (m)  Right Precuneus, parieto-occipital sulcus (dm) |
| 3 | Right posterior Superior Temporal Sulcusc (cp) Right Inferior Parietal Lobule BA40 (rv) | 23 | Left Superior Frontal Gyrus BA8 (m) Right Superior Frontal Gyrus BA8 (m) Left Superior Frontal Gyrus BA8 (dl)  Right Superior Frontal Gyrus BA6 (m) | 43 | Right Superior Frontal Gyrus BA6 (dl) Right Middle Frontal Gyrus, BA6 (vl) Right Precentral Gyrus BA6 (cdl) Right Precentral Gyrus BA4 (ul) | 63 | Right Middle Temporal Gyrus, temporal sulcus (as) | 83 | Left Superior Temporal Gyrus BA38 (l) Left Middle Temporal Gyrus BA21 (r) Left Insular Gyrus (vId/vIg) Left Basal Ganglia, putamen (dl) Left pre-motor thalamus |
| 4 | Right Superior Temporal Gyrus BA38 (l) Right Middle Temporal Gyrus BA21 (r) Right Insular Gyrus (vId/vIg) | 24 | Right Superior Temporal Gyrus BA38 (m)  Right Amygdala (l) | 44 | Right Inferior Frontal Gyrus BA44 (d) Right Inferior Frontal Gyrus BA45 (c) Right Inferior Frontal Gyrus BA44 (v) Right Precentral Gyrus BA4 (tla) | 64 | Right Basal Ganglia, caudate (v) Right medial pre-frontal thalamus | 84 | Right lateral Occipital Cortex, occipital polar cortex Right lateral Occipital Cortex, inferior occipital gyrus |
| 5 | Right rostraltemporal Thalamus | 25 | Left Superior Parietal Lobule BA5 (l) Left Superior Parietal Lobule BA7 (ip)  Left Postcentral Gyrus BA2 | 45 | Left Inferior Temporal Gyrus BA20 (r) Left Fusiform Gyrus BA20 (rv)  Left Parahippocampal Gyrus BA35/36 (r) Left Parahippocampal Gyrus BA28/34 entorhinal cortex | 65 | Right Basal Ganglia, caudate (d) Right caudal temporal thalamus | 85 | Right Orbital Gyrus BA12/47 (l) Right Insular Gyrus (dIa) |
| 6 | Left Superior Temporal Gyrus, BA22 (r) Left Middle Temporal Gyrus, STS (a) Left Inferior Temporal Gyrus, BA20 (cv) | 26 | Left Precuneus BA7 (m) Left Precuneus BA5 (m) | 46 | Left sensory thalamus Left posterior parietal thalamus | 66 | Left Inferior Temporal Gyrus BA20 (iv/il) Left Parahippocampal Gyrus BA35/36 (c) Left Parahippocampal Gyrus (lp) Left hippocampus (c) | 86 | Left Orbital Gyrus BA13 Left Basal Ganglia, caudate (v) |
| 7 | Right Middle Frontal Gyrus BA46 Right Middle Frontal Gyrus BA10 (l) | 27 | Right Superior Temporal Gyrus, BA41/42 Right Superior Temporal Gyrus BA22 (c)  Right Postcentral Gyrus BA1/2/3 (tla) | 47 | Right Inferior Parietal Lobule, BA39 (rd) Right lateral Occipital Cortex, lateralsuperior occipital gyrus | 67 | Left Superior Frontal Gyrus BA6 (m) Left Paracentral Lobule BA1/2/3, BA4 (ll)  Right Paracentral Lobule A4 (ll)  Left Cingulate Gyrus BA23 (c ) | 87 | Left Basal GangliadCa, caudate (d) Left rostral temporal thalamus |
| 8 | Left Medioventral Occipital Cortex, cuneus gyrus (r) Left Medioventral Occipital Cortexc, cuneus gyrus (c) Left lateral Occipital Cortex, occipital polar cortex | 28 | Left Basal Ganglia, globuspallidus Left Thalamusm medialpre-frontal Left Thalamus, lateralpre-frontal | 48 | Right Paracentral Lobule BA1/2/3 (ll) Right Precuneus, BA5 (m) | 68 | Left Superior Temporal Gyrus BA41/42  Left Superior Temporal Gyrus BA22 (c) Left Inferior Parietal Lobule BA40 (rv) | 88 | Left Precentral Gyrus BA4 (hf) Left Precentral Gyrus BA6 (cvl) |
| 9 | Left Middle Temporal Gyrus, BA21 (c) Left Inferior Temporal Gyrus, BA20 (cl) Left Fusiform Gyrus, BA37 (lv) | 29 | Left Inferior Frontal Gyrus, BA44 (d) | 49 | Right medial Parahippocampal Gyrus  Right occipital Thalamus | 69 | Right Inferior Parietal Lobule BA39 (c) Right lateral Occipital Cortex, middle occipital gyrus | 89 | Left Cingulate Gyrus BA23 (v) |
| 10 | Left Precuneus dm parietooccipital sulcus (PEr) Left Precuneus BA31 (Lc1) Left Cingulate Gyrus, BA23 (d) | 30 | Left+right Cingulate Gyrus, BA24 (cd) Right Cingulate Gyrus, BA23 (c) | 50 | Left Inferior Frontal Gyrus BA44 (v) Left Insular Gyrus (dId) | 70 | Right Precuneus, BA31 (Lc1) Right Cingulate Gyrus BA23 (d)  Right Cingulate Gyrus BA23 (v) | 90 | Right Superior Temporal Gyrus BA22 (c) |
| 11 | Left Superior Frontal Gyrus BA10 (m) Right Superior Frontal Gyrus BA10 (m) Left + Right Orbital Gyrus BA11 (m) Left Cingulate Gyrus BA32 (sg) | 31 | Left Inferior Frontal Gyrus BA45 (r) Left Inferior Frontal Gyrus BA44 (op) | 51 | Right Inferior Parietal Lobule, BA40 (c)  Right Inferior Parietal Lobule, BA39 (rv) | 71 | Left Superior Parietal Lobule BA7 (pc)  Left Inferior Parietal Lobule BA39 (rd) | 91 | Right Precentral Gyrus BA4 (t) |
| 12 | Left Middle Frontal Gyrus, BA46 | 32 | Right Parahippocampal Gyrus BA35/36 (c) Right lateroposterior Parahippocampal Gyrus Right Hippocampus (c) | 52 | Left Precentral Gyrus BA6 (cdl)  Left Precentral Gyrus BA4 (ul)  Left Postcentral Gyrus BA1/2/3 (tru) | 72 | Right sensory thalamus Right posterior parietal thalamus | 92 | Left Middle Frontal Gyrus BA9/46 (v) Left Inferior Frontal Gyrus, inferior frontal sulcus |
| 13 | Right Superior Frontal Gyrus BA9 (l)  Left Orbital Gyrus BA14 (m) | 33 | Right Parahippocampal Gyrus BA 28/34, entorhinal cortex  Right Parahippocampal Gyrus, insular cortex (ta) Right Amygdala (m) | 53 | Right Superior Temporal Gyrus (TE1.0andTE1.2) Right Insular Gyrus, insula (dg / dlg) Right Basal Ganglia, globus pallidus Right pre-motor thalamus | 73 | Left Superior Parietal Lobule BA7 (r) Left Superior Parietal Lobule BA7 © | 93 | Right Insular Gyrus (vIa) Right Basal Ganglia, putamen (vm) Right Basal Ganglia, putamen (dl) |
| 14 | Left Amygdala (l)  Left Hippocampus (r) | 34 | Right Middle Temporal Gyrus BA37 (dl) Right Inferior Temporal Gyrus BA37 (vl) Right posterior Superior Temporal Sulcus (r) Right lateral Occipital Cortex, V5/MT+ | 54 | Right Fusiform Gyrus BA20 (rv) | 74 | Right Insular Gyrus (hg) Right lateral pre-frontal thalamus | 94 | Left Inferior Parietal Lobule BA39 (c) Left Inferior Parietal Lobule BA39 (rv) |
| 15 | Left Precentral Gyrus, BA4 (tla)  Left Superior Temporal Gyrus (TE1.0 / TE1.2) Left Postcentral Gyrus BA1/2/3 (tIa) Left Insular Gyrus (hg/dg) | 35 | Right Superior Frontal Gyrus, BA8 (dl) Right Middle Frontal Gyrus, BA9/46 (d) Right Middle Frontal Gyrus, BA8 (vl) | 55 | Left Medioventral Occipital Cortexc, lingual gyrus (c) Left Medioventral Occipital Cortex lingualgyrus (r) | 75 | Right Superior Parietal Lobule BA5 (l) Right Postcentral Gyrus BA1/2/3 (tru) | 95 | Left lateral Occipital Cortex, occipital gyrus (m) Left lateral Occipital Cortex, V5/MT+ Left lateral Occipital Cortex, occipital gyrus (i) |
| 16 | Right Middle Temporal Gyrus, BA21 (c) Right Inferior Temporal Gyrus, BA20 (cl) Right Inferior Temporal Gyrus, BA20 (cv) | 36 | Left Inferior Parietal Lobule BA40 (rd) Left Inferior Parietal Lobule BA40 (c) Left Postcentral Gyrus BA1/2/3 (ulhf) | 56 | Right Orbital Gyrus BA12/47 (o) Right Orbital Gyrus BA11 (l) | 76 | Right Superior Parietal Lobule BA7 (r) Right Superior Parietal Lobule BA7 (pc) Right Superior ParietalLobule BA7 (ip) | 96 | Left Precentral Gyrus BA4 (t) |
| 17 | Left Superior Temporal Gyrus, BA38 (m) Left Parahippocampal Gyrus, temporal insular cortex (ag) Left Insular Gyrus (vla/dla) Left Amygdala (m) Left Basal Ganglia, nucleus acc., putamen (vm) | 37 | Right Middle Frontal Gyrus, inferior frontal junction Right Precentral Gyrus BA6 (cvl) | 57 | Left Superior Frontal Gyrus, BA9 (l) Left Superior Frontal Gyrus, BA9 (m) Right Superior Frontal Gyrus, BA9 (m) | 77 | Left Medioventral occipital Cortex, parieto-occipitalsulcus (vm) | 97 | Left Inferior Frontal Gyrus BA45 ( c ) |
| 18 | Left lateral Occipital Cortex, occipitalgyrus (ms) | 38 | Right Middle Frontal Gyrus A9/46 (v) Right Inferior Frontal Gyrus IFS, BA44 (op), BA45 ( c ) | 58 | Right Inferior Temporal Gyrus BA37, (elv) Right Fusiform Gyrus, BA37 (mv+lv) | 78 | Left Middle Temporal Gyrus BA37 (dl) Left InferiorTemporal Gyrus BA37 (elv+lv) | 98 | Right Inferior Temporal Gyrus BA20 (r) Right Parahippocampal Gyrus BA35/36 (r) |
| 19 | Right Medio Ventral Occipital Cortex, cuneus gyrus (r) Right Medio Ventral Occipital Cortex, parieto-occipital sulcus (vm) | 39 | Left Cingulate Gyrus BA24 (rv) Left Cingulate Gyrus A32 (pg) | 59 | Left lateral Occipital Cortex, occipitalgyrus (ls) | 79 | Left Parahippocampal Gyrus | 99 | Left Superior Temporal Sulcus (rp) Left SuperiorTemporalSulcus (cp) |
| 20 | Right Inferior Temporal Gyrus, BA20 (iv) Right Inferior Temporal Gyrus, BA20 (il) | 40 | Right Superior Parietal Lobule A7 (c) Right lateral Occipital Cortex, occipital gyrus (ms) | 60 | Left Fusiform Gyrus, BA37 (mv) | 80 | Left Middle Frontal Gyrus BA10 (l) | 100 | Right Medio Ventral Occipital Cortex, lingual gyrus (c) Right Medio Ventral Occipital Cortex, cuneus gyrus (c) |
|  |  |  |  |  |  |  |  |  |  |

***Figure 1 - 2.*** *Table showing the parcel identities with anatomical labeling derived from the BNA atlas(Jiang, 2013). Mention of several areas means the parcel has been created by merging these originally separate BNA parcels into a single parcel. Abbreviations: m = medial, l = lateral, r = rostral, a = anterior, p = posterior, v = ventral, c = caudal, i = inferior, o = orbital, p = pregenual, ag = agranular, rv = rostroventral, sg = subgenual, cv = caudoventral, cl = caudolateral, dg = dorsal granular, lv = lateroventral, dm = dorsomedial, vm = ventromedial, tla = tongue and larynx area, ms = medial superior, iv = intermediate ventral, rd = rostrodorsal, cd = caudodorsal, rp = rostroposterior, op = opercular, vla/d = ventral a/dysgranular, da = dorsal agranular, ms = medial superior, ulhf = upper limb, head and face region, op = opercular, pg= pregenual, ms = medial superior, ll = lower limb region, dld = dorsal dysgranular, iv = intermediate ventral, pc = postcentral, ip = intraparietal, vld = ventraldysgranular, vlg = ventral granular, dla = dorsal agranular, cvl = caudal ventral lateral, ta = temporal agranular, lp = lateral posterior.*
